# A Comparative Evaluation Between Dermatoglyphics and Canine Relationship in Deciduous Dentition: An Analysis for Prediction

**DOI:** 10.7759/cureus.69802

**Published:** 2024-09-20

**Authors:** Harini M, Vignesh Ravindran, Abirami Arthanari

**Affiliations:** 1 Department of Pediatric Dentistry, Saveetha Dental College and Hospitals, Saveetha Institute of Medical and Technical Sciences, Saveetha University, Chennai, IND; 2 Department of Forensic Odontology, Saveetha Dental College and Hospitals, Saveetha Institute of Medical and Technical Sciences, Saveetha University, Chennai, IND

**Keywords:** dental health, dermatoglyphics, genetic traits, pediatric dentistry, primary canine relationships

## Abstract

Objective: This study aimed to investigate the potential correlation between dermatoglyphic patterns and primary canine relationships in children aged three to six years. Dermatoglyphics, the study of ridge patterns on fingers, has been linked to various genetic and developmental conditions. Similarly, primary canine relationships provide insights into dental health and potential orthodontic needs.

Materials and methods: A cross-sectional study was conducted involving 600 children with complete primary dentition. Dermatoglyphic patterns were recorded using the ink and roller method, and primary canine relationships were assessed through clinical examination and dental cast analysis. Patterns were classified into arches, loops, and whorls, while canine relationships were categorized into Class I, II, or III. Statistical analyses, including chi-square tests and correlation coefficients, were performed to explore potential associations.

Results: The study found no significant correlation between dermatoglyphic patterns and primary canine relationships across various fingers of both hands. For each finger, the distribution of arches, loops, and whorls did not differ significantly among Classes I, II, and III canine relationships, with p-values ranging from 0.107 to 0.977.

Discussion: The results indicate that dermatoglyphic patterns and primary canine relationships are not directly correlated in this sample. This aligns with previous research suggesting that while both traits are influenced by genetic factors, their direct interaction may be less straightforward than hypothesized. The study highlights the complexity of genetic and developmental influences on dental and dermatoglyphic traits and underscores the need for further research.

Conclusion: This study found no significant association between dermatoglyphic patterns and primary canine relationships. Future research should employ longitudinal designs and include diverse populations to explore indirect correlations and interactions. Understanding these relationships may enhance early diagnostic and predictive practices in pediatric dentistry, contributing to improved child health outcomes.

## Introduction

The exploration of dermatoglyphic patterns has long provided valuable insights into genetic and developmental anomalies, contributing significantly to fields such as forensic science, anthropology, and medicine. Dermatoglyphics, the study of ridge patterns on the fingers, palms, toes, and soles, is known for its use in personal identification and its potential to indicate genetic disorders [1]. These unique patterns are established early in fetal development and remain unchanged throughout life, serving as a permanent record of embryonic development. Various studies have demonstrated that dermatoglyphic patterns can be associated with specific medical conditions, highlighting their diagnostic value in predicting genetic syndromes [2-4].

In parallel, the evaluation of canine relationships in primary dentition provides critical information about dental health and future orthodontic needs. The position and occlusion of primary canines are key indicators of normal dental development and are used to predict malocclusions in permanent teeth. Proper alignment of the primary canines is essential for guiding the eruption and placement of permanent teeth, thus playing a crucial role in maintaining overall oral health. Deviations from normal canine relationships in primary dentition can signal potential dental issues that may require early intervention [5].

Recent research has begun to investigate potential correlations between dermatoglyphic patterns and dental conditions, including malocclusions and dental caries. While significant associations have been found between certain dermatoglyphic traits and the prevalence of dental issues, studies specifically comparing these patterns with canine relationship patterns in primary dentition are limited [6]. For instance, research evaluating cheiloscopic patterns and their relation to primary canine relationships in children has shown promising results, suggesting that non-invasive dermatoglyphic analysis could be utilized for early prediction and management of dental health problems [7].

The rationale for investigating the relationship between dermatoglyphic patterns and canine relationships in primary dentition lies in the shared genetic and developmental origins of these traits. Both dermatoglyphic patterns and dental structures are influenced by genetic factors and develop concurrently during fetal life [8]. Understanding these connections could lead to improved diagnostic tools and preventive measures in pediatric dentistry. This study aims to explore the potential correlations between dermatoglyphic patterns and primary canine relationships, to enhance early detection and intervention strategies for dental malocclusions and other related conditions.

## Materials and methods

Study design and approval

This cross-sectional study aimed to explore the correlation between dermatoglyphic patterns and primary canine relationships in a sample of 600 children aged between three and five years. Prior to initiating the study, ethical approval was obtained from the institutional review board (SRB/SDC/UG/Pedo/2024/02/043). Data collection was carried over the period of February 2024 to July 2024.

Study population and standardization

The study included 600 children with complete primary dentition, i.e., less than five years of age. Inclusion criteria were children aged three to five years with complete primary dentition with no signs of changes related to the first transitional period. Exclusion criteria included children with craniofacial anomalies, a history of dental extraction, children with prolonged deleterious oral habits, previous dental trauma, or systemic conditions affecting growth and development. For standardization of the population in order to completely attribute the results to the dermatoglyphic patterns and to avoid bias based on gender selection and canine relation, 200 children per canine classification were recruited among which 100 were males and 100 were females.

Informed consent

The study’s purpose, procedures, potential risks, and benefits were explained in detail to ensure voluntary participation. Participants were assured of their right to withdraw from the study at any time without any consequences. Informed consent was obtained from the parents or guardians of all participating children.

Population recruitment

A comprehensive clinical examination was performed among children attending the dental outpatient department of a private dental institute to assess the primary canine relationships. All children were examined until the required sample size was met. The dental examination involved using a mouth mirror, explorer, and dental probe to evaluate the alignment and occlusion of primary canines. 

Primary canine relationship assessment

The canine relationship was classified into Class I, Class II, or Class III based on the relative positions of the maxillary and mandibular primary canines. Class I canine relationship is when the cusp tip of the maxillary primary canine aligns with the embrasure space between the mandibular primary canine and the first primary molar. Class II canine relationship is when the cusp tip of the maxillary primary canine is positioned mesial to the embrasure space between the mandibular primary canine and the first primary molar. Class III canine relationship is when the cusp tip of the maxillary primary canine is positioned distal to the embrasure space between the mandibular primary canine and the first primary molar.

Dermatoglyphic pattern analysis

**Figure 1:**
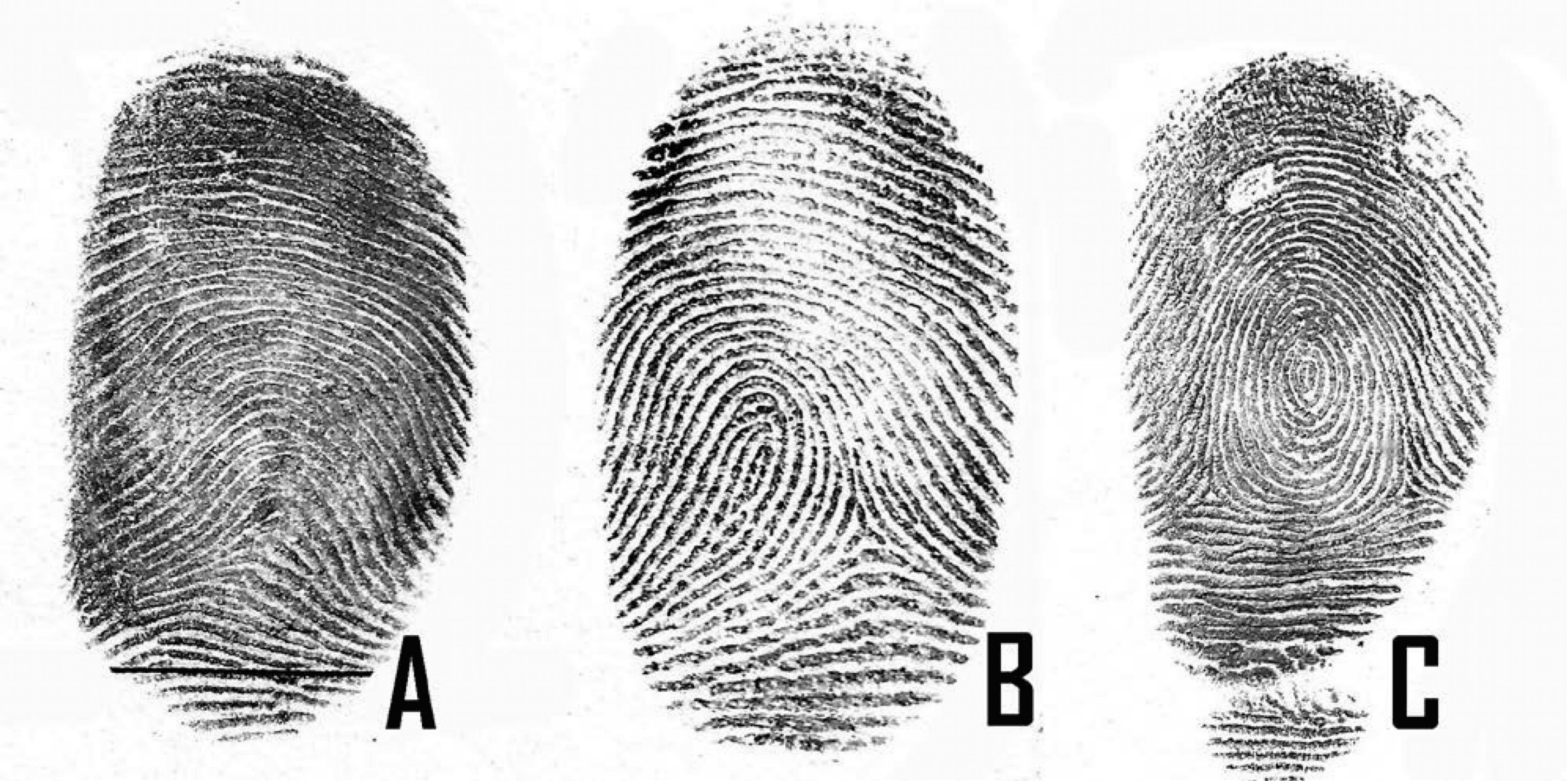
Dermatoglyphic patterns for assessment in the present study: arch (A), loop (B), and whorl (C).

The dermatoglyphic patterns were recorded using the ink and roller method suggested by Cummins and Midlo [9]. An ink pad and roller were used to apply a thin layer of ink evenly on the mixing pad. Each child’s fingerprints were then carefully rolled from one side of the nail to the other on a clean white paper to capture complete ridge patterns. The prints were allowed to dry and subsequently scanned using a high-resolution scanner for detailed analysis. Dermatoglyphic patterns were classified into loops, whorls, and arches using a magnifying glass by a forensic odontologist (Figures 1A-1C).

Data analysis

Statistical analysis was conducted using SPSS version 23.0 (IBM Corp., Armonk, NY). Chi-square tests were employed to analyze the relationships between dermatoglyphic patterns and canine relationships. Multiple regression analysis was performed to control for potential confounding variables and determine the predictive value of dermatoglyphic patterns for canine relationships. p < 0.05 is considered to be statistically significant.

## Results

In this study, we examined the correlation between dermatoglyphic patterns (arches, loops, and whorls) and primary canine relationships (Classes 1, 2, and 3) across different fingers of both hands. For the left thumb, 18 children (9%) with Class 1 canine relationships had arch patterns, 113 children (57%) had loop patterns, and 69 children (35%) had whorl patterns. Similar distributions were observed in Class 2 and Class 3, with no significant difference among the groups (p-value = 0.977). For the left forefinger, 41 children (21%) in Class 1 had arch patterns, 93 children (47%) had loops, and 66 children (33%) had whorls, with no significant association found (p-value = 0.402) (Table 1).

**Table 1:** Dermatoglyphic pattern distribution among the participants of the study.

Canine relation	Arch	Loop	Whorl	P-value
Left thumb				
1	18 (9%)	113 (57%)	69 (35%)	0.977
2	18 (9%)	112 (56%)	70 (35%)	
3	19 (10%)	117 (59%)	64 (32%)	
Left fore				
1	41 (21%)	93 (47%)	66 (33%)	0.402
2	28 (14%)	98 (49%)	74 (37%)	
3	34 (17%)	88 (44%)	78 (39%)	
Left middle				
1	19 (10%)	126 (63%)	55 (28%)	0.763
2	19 (10%)	117 (59%)	64 (32%)	
3	16 (8%)	118 (59%)	66 (33%)	
Left ring				
1	6 (3%)	87 (44%)	107 (54%)	0.207
2	9 (5%)	75 (38%)	116 (58%)	
3	15 (8%)	74 (37%)	111 (56%)	
Left little				
1	16 (8%)	128 (64%)	56 (28%)	0.329
2	13 (7%)	119 (60%)	68 (34%)	
3	16 (8%)	135 (68%)	49 (25%)	
Right thumb				
1	20 (10.0%)	114 (57.0%)	66 (33.0%)	0.107
2	25 (12.5%)	97 (48.5%)	78 (39.0%)	
3	19 (9.5%)	124 (62.0%)	57 (28.5%)	
Right fore				
1	24 (12.0%)	101 (50.5%)	75 (37.5%)	0.813
2	24 (12.0%)	107 (53.5%)	69 (34.5%)	
3	23 (11.5%)	113 (56.5%)	64 (32.0%)	
Right middle				
1	13 (6.5%)	145 (72.5%)	42 (21.0%)	0.72
2	12 (6.0%)	154 (77.0%)	34 (17.0%)	
3	14 (7.0%)	142 (71.0%)	44 (22.0%)	
Right ring				
1	9 (4.5%)	86 (43.0%)	105 (52.5%)	0.895
2	8 (4.0%)	85 (42.5%)	107 (53.5%)	
3	6 (3.0%)	81 (40.5%)	113 (56.5%)	
Right little				
1	12 (6.0%)	137 (68.5%)	51 (25.5%)	0.69
2	13 (6.5%)	140 (70.0%)	47 (23.5%)	
3	8 (4.0%)	148 (74.0%)	44 (22.0%)	

In the left middle finger, Class 1 had 19 children (10%) with arches, 126 children (63%) with loops, and 55 children (28%) with whorls. The distributions in Class 2 and Class 3 were similar, with p-value = 0.763 indicating no significant correlation. For the left ring finger, Class 1 had six children (3%) with arches, 87 children (44%) with loops, and 107 children (54%) with whorls. The distributions for Class 2 and Class 3 showed no significant differences (p-value = 0.207) (Table 1).

For the right thumb, Class 1 had 20 children (10%) with arches, 114 children (57%) with loops, and 66 children (33%) with whorls. Class 2 and Class 3 had similar patterns, with no significant difference (p-value = 0.107). On the right forefinger, the distributions were 24 children (12%) with arches, 101 children (50.5%) with loops, and 75 children (37.5%) with whorls in Class 1. Class 2 and Class 3 distributions showed no significant correlation (p-value = 0.813) (Table 1).

The right middle finger showed Class 1 with 13 children (6.5%) with arches, 145 children (72.5%) with loops, and 42 children (21%) with whorls, with similar distributions in Class 2 and Class 3 (p-value = 0.72). For the right ring finger, Class 1 had nine children (4.5%) with arches, 86 children (43%) with loops, and 105 children (52.5%) with whorls. The patterns in Class 2 and Class 3 showed no significant differences (p-value = 0.895). Finally, on the right little finger, Class 1 had 12 children (6%) with arches, 137 children (68.5%) with loops, and 51 children (25.5%) with whorls, with no significant association found across the classes (p-value = 0.69). Overall, the results indicate no significant correlation between dermatoglyphic patterns and primary canine relationships across the different fingers (Table 1). Multiple regression analysis did not provide any significant correlations as none of the patterns had an interconnected association with the above analysis (p>0.05).

## Discussion

The rationale behind this study was to explore the potential correlation between dermatoglyphic patterns and primary canine relationships in children aged three to six years. Dermatoglyphics, the study of the intricate patterns of ridges on fingers, palms, and soles, has been linked to genetic and developmental conditions [10]. Similarly, the alignment and relationship of primary canines can provide early indicators of dental and orthodontic issues. Given these potential connections, our study aimed to investigate whether a significant correlation exists between these two factors, providing a novel approach to early diagnosis and prediction of dental anomalies.

Our methodology involved a thorough collection and analysis of dermatoglyphic patterns using the ink and roller method recommended by Cummins and Midlo, which ensured high-quality and detailed prints for analysis. This method has been extensively validated in previous studies for its accuracy and reliability in capturing fingerprint patterns [11]. Additionally, the classification of primary canine relationships was performed through clinical examination and dental cast analysis, a standard practice in pediatric dentistry to ensure precise assessment of dental occlusion [12].

Our results demonstrated no significant correlation between dermatoglyphic patterns (arches, loops, and whorls) and primary canine relationships (Classes 1, 2, and 3) across different fingers. These findings are consistent with previous studies that have explored similar associations. For instance, research by Mathew et al. and Reddy et al. did not find significant correlations between fingerprint patterns and dental anomalies, suggesting that while dermatoglyphics can reflect genetic and developmental factors, their direct relationship with specific dental traits remains unclear [13,14].

Despite the lack of significant findings, this study adds to the body of knowledge by reaffirming the complexity of genetic and developmental influences on dental and dermatoglyphic traits. The results suggest that while both dermatoglyphic patterns and primary canine relationships are influenced by genetic factors, their interaction may be more intricate and less direct than previously hypothesized. This creates the need for further research with larger sample sizes and more diverse populations to explore potential indirect or multifactorial correlations [15].

In comparing our findings with previous research, it is clear that the relationship between dermatoglyphic patterns and dental traits is still an emerging field with many unanswered questions. Other published research has shown that while there are genetic correlations, the phenotypic expressions in fingerprints and dental structures may not be straightforwardly connected [16,17]. This suggests a complex interplay of genetic, epigenetic, and environmental factors that warrant further investigation.

One of the key strengths of this study was the large sample size of 600 children, which provided a robust data set for statistical analysis. Additionally, the use of standardized and validated methods for both dermatoglyphic and dental assessments ensured the reliability and validity of our findings. The study's limitations include its cross-sectional design, which restricts the ability to infer causal relationships between dermatoglyphic patterns and primary canine relationships. Additionally, the sample population was relatively homogeneous in terms of age and ethnicity, potentially limiting the generalizability of the findings to more diverse populations. The ink and roller method, while reliable, may introduce variability based on the examiner's skill and the child's cooperation. Furthermore, the study did not account for potential genetic and environmental factors that could influence both dermatoglyphic patterns and dental traits, warranting further exploration through longitudinal studies.

Future research should build upon these results by employing longitudinal study designs and including more diverse populations to explore potential indirect correlations and interactions over time. Incorporating genetic and epigenetic factors could further elucidate the complex interplay between dermatoglyphics and dental traits. This continued exploration is crucial for refining diagnostic tools and predictive measures in pediatric dentistry, ultimately contributing to better health outcomes for children.

## Conclusions

In conclusion, this study did not find a significant correlation between dermatoglyphic patterns and primary canine relationships in children. While this might initially seem discouraging, it highlights the importance of continuing research in this area to uncover the underlying mechanisms that may link these traits. By understanding these connections better, we can improve early diagnostic and predictive practices in pediatric dentistry, ultimately leading to better health outcomes for children.
